# Supplementary material for: Effectiveness of virtual reality technology in rehabilitation after anterior cruciate ligament reconstruction: A systematic review and meta-analysis
Source: PLoS One. 2025 Mar 3;20(3):e0314766. doi: 10.1371/journal.pone.0314766 (PMC11875343; doi:10.1371/journal.pone.0314766)
Supplement: S1 File — (PDF) [file pone.0314766.s002.pdf]

| Literature Library | Search queries | Search results |
|---|---|---|
| PubMed | #1 "Anterior Cruciate Ligament"[Mesh] | 12,666 |
| | #2 ((((((((((((((Anterior Cruciate Ligament Reconstruction[Title/Abstract]) OR (Anterior Cruciate Ligament Injury[Title/Abstract])) OR (Cruciate Ligament, Anterior[Title/Abstract])) OR (Anterior Cruciate Ligaments[Title/Abstract])) OR (Cruciate Ligaments, Anterior[Title/Abstract])) OR (Ligament, Anterior Cruciate[Title/Abstract])) OR (Ligaments, Anterior Cruciate[Title/Abstract])) OR (Anterior Cranial Cruciate Ligament[Title/Abstract])) OR (Cranial Cruciate Ligament[Title/Abstract])) OR (Cranial Cruciate Ligaments[Title/Abstract])) OR (Cruciate Ligament, Cranial[Title/Abstract])) OR (Cruciate Ligaments, Cranial[Title/Abstract])) OR (Ligament, Cranial Cruciate[Title/Abstract])) OR (Ligaments, Cranial Cruciate[Title/Abstract])) OR (ACL[Title/Abstract])) OR (ACLR[Title/Abstract]) | 27,453 |
| | #3= #1 OR #2 | 30,056 |
| | #4 "Virtual Reality"[Mesh] | 6,495 |
| | #5 ((((((((Reality, Virtual[Title/Abstract]) OR (VR[Title/Abstract])) OR (Virtual Environment[Title/Abstract])) OR (Virtual Rehabilitation[Title/Abstract])) OR (Immersive Multimedia[Title/Abstract])) OR (Computer-simulated Reality[Title/Abstract])) OR (Video Game[Title/Abstract])) OR (Virtual Game[Title/Abstract])) OR (Virtual Therapy[Title/Abstract]) | 19,999 |
| | #6= #4 OR #5 | 23,175 |
| | #7= #3 AND #6 | 24 |
| Web of Science | #1 (((((((((((((((TS=(Anterior Cruciate Ligament )) OR TS=(Anterior Cruciate Ligament Reconstruction)) AND TS=(Anterior Cruciate Ligament Injury)) OR TS=(Cruciate Ligament, Anterior)) OR TS=(Anterior Cruciate Ligaments)) OR TS=(Cruciate Ligaments, Anterior)) OR TS=(Ligament, Anterior Cruciate)) OR TS=(Ligaments, Anterior Cruciate)) OR TS=(Anterior Cranial Cruciate Ligament)) OR TS=(Cranial Cruciate Ligament)) OR TS=(Cranial Cruciate Ligaments)) OR TS=(Cruciate Ligament, Cranial)) OR TS=(Cruciate Ligaments, Cranial)) OR TS=(Ligament, Cranial Cruciate)) OR TS=(Ligaments, Cranial Cruciate)) OR TS=(ACL)) OR TS=(ACLR) and Preprint Citation Index (Exclude – Database) | 52,625 |
| | #2 (((((((((TS=(Virtal Reality)) OR TS=(Reality, Virtual)) OR TS=(VR )) OR TS=(Virtual Environment )) OR TS=(Immersive Multimedia)) OR TS=(Virtual Rehabilitation)) OR TS=(Computer-simulated Reality)) OR TS=(Video Game )) OR TS=(Virtual Game )) OR TS=(Virtual Therapy) and Preprint Citation Index (Exclude – Database) | 354,899 |
| | #3= #1 AND #2 | 179 |
| Cochrane Library | #1 MeSH descriptor: [Anterior Cruciate Ligament] explode all trees | 846 |
| | #2 (Anterior Cruciate Ligament Reconstruction):ti,ab,kw OR (Anterior Cruciate Ligament Injury):ti,ab,kw OR (Cruciate Ligament, Anterior):ti,ab,kw OR (Anterior Cruciate Ligaments):ti,ab,kw OR (Cruciate Ligaments, Anterior):ti,ab,kw OR (Ligament, Anterior Cruciate):ti,ab,kw OR (Ligaments, Anterior Cruciate):ti,ab,kw OR (Anterior Cranial Cruciate Ligament):ti,ab,kw OR (Cranial Cruciate Ligament):ti,ab,kw OR (Cranial Cruciate Ligaments):ti,ab,kw OR (Cruciate Ligament, Cranial):ti,ab,kw OR (Cruciate Ligaments, Cranial):ti,ab,kw OR (Ligament, Cranial Cruciate):ti,ab,kw OR(Ligaments, Cranial Cruciate):ti,ab,kw OR (ACL):ti,ab,kw OR (ACLR):ti,ab,kw | 4,126 |
| | #3= #1 OR #2 | 4,126 |
| | #4 MeSH descriptor: [Virtual Reality] explode all trees | 1,094 |
| | #5 (Reality,Virtual):ti,ab,kw OR (VR):ti,ab,kw OR (Virtual Environment):ti,ab,kw OR (Virtual Rehabilitation):ti,ab,kw OR (Immersive Multimedia):ti,ab,kw OR (Computer-simulated Reality):ti,ab,kw OR (Video Game ):ti,ab,kw OR (Virtual Game ):ti,ab,kw OR (Virtual Therapy):ti,ab,kw | 10,420 |
| | #6= #4 OR #5 | 10,595 |
| | #7= #3 AND #6 | 17 |
| EMbase | #1 'anterior cruciate ligament'/exp OR 'anterior cruciate ligament' | 37,627 |
| | #2 'anterior cruciate ligament reconstruction':ab,ti OR 'anterior cruciate ligament injury':ab,ti OR 'cruciate ligament, anterior':ab,ti OR 'anterior cruciate ligaments':ab,ti OR 'cruciate ligaments, anterior':ab,ti OR 'ligament, anterior cruciate':ab,ti OR 'ligaments, a | 34,509 |

| | | |
|---|---|---|
| | nterior cruciate':ab,ti OR 'anterior cranial cruciate ligament':ab,ti OR 'cranial cruciate ligament':ab,ti OR 'cranial cruciate ligaments':ab,ti OR 'cruciate ligament, cranial':ab,ti OR 'cruciate ligaments, cranial':ab,ti OR 'ligament, cranial cruciate':ab,ti OR 'ligaments, cranial cruciate':ab,ti OR 'acl':ab,ti OR 'aclr':ab,ti | |
| | #4 'virtual reality'/exp OR 'virtual reality' | 36,956 |
| | #5 'reality, virtual':ab,ti OR 'vr':ab,ti OR 'virtual environment':ab,ti OR 'virtual rehabilitation':ab,ti OR 'immersive multimedia':ab,ti OR 'computer-simulated reality':ab,ti OR 'video game':ab,ti OR 'virtual game':ab,ti OR 'virtual therapy':ab,ti | 25,884 |
| | #6= #4 OR #5 | 51,734 |
| | #7= #3 AND #6 | 81 |
| EBSCO | #1 SU Anterior Cruciate Ligament OR SU Anterior Cruciate Ligament Reconstruction OR SU Anterior Cruciate Ligament Injury OR SU Cruciate Ligament, Anterior OR SU Anterior Cruciate Ligaments OR SU Cruciate Ligaments, Anterior OR SU Ligament, Anterior Cruciate OR SU Ligaments, Anterior Cruciate OR SU Anterior Cranial Cruciate Ligament OR SU Cranial Cruciate Ligament OR SU Cranial Cruciate Ligaments OR SU Cruciate Ligament, Cranial | 37,114 |
| | #2 SU Cruciate Ligaments, Cranial OR SU Ligament, Cranial Cruciate OR SU Ligaments, Cranial Cruciate OR SU ACL OR SU ACLR | 3,744 |
| | #3= #1 OR #2 | 38,154 |
| | #4 SU Virtal Reality OR SU Reality, Virtual OR SU VR OR SU Virtual Environment OR SU Virtual Rehabilitation OR SU Immersive Multimedia OR SU Computer-simulated Reality OR SU Video Game OR Virtual Game OR Virtual Therapy | 96,189 |
| | #5= #3 AND #4 | 27 |
| CNKI | （主题：前交叉韧带）OR（主题：前交叉韧带重建）OR（主题：前交叉韧带损伤）OR（主题：ACL）OR（主题：ACLR）AND（主题：虚拟现实）OR（主题：VR）OR（主题：虚拟环境）OR（主题：虚拟现实技术） | 13 |
| CBM | #1 "前交叉韧带"[不加权:扩展] | 4,404 |
| | #2 "前交叉韧带重建"[不加权:扩展] | 1,658 |
| | #3 "前交叉韧带损伤"[不加权:扩展] | 1,362 |
| | #4 "ACL"[常用字段:智能] | 3,820 |
| | #5 "ACLR"[常用字段:智能] | 96 |
| | #6= (#5) OR (#4) OR (#3) OR (#2) OR (#1) | 7,221 |
| | #7 "虚拟现实"[不加权:扩展] | 1,984 |
| | #8 "VR"[常用字段:智能] | 6,370 |
| | #9 "虚拟环境"[常用字段:智能] | 265 |
| | #10 "虚拟现实技术"[常用字段:智能] | 1,733 |
| | #11= (#10) OR (#9) OR (#8) OR (#7) | 8,291 |
| | #12= (#11) AND (#6) | 14 |
| VIP | (((((((((题名或关键词=十字韧带 OR 题名或关键词=前十字韧带) OR 题名或关键词=伸肌下支持带) OR 题名或关键词=膝交叉韧带) OR 题名或关键词=前交叉韧带) OR 题名或关键词=小腿十字韧带) OR 题名或关键词=前交叉韧带重建) OR 题名或关键词=前交叉韧带损伤) OR 题名或关键词=ACL) OR 题名或关键词=ACLR) AND (((题名或关键词=虚拟现实 OR 题名或关键词=VR) OR 题名或关键词=虚拟环境) OR 题名或关键词=虚拟现实技术)) | 10 |
| WanFang | (主题=(前交叉韧带 OR 前交叉韧带重建 OR 前交叉韧带损伤 OR ACL OR ACLR)) AND 主题=(虚拟现实 OR VR OR 虚拟环境 OR 虚拟现实技术) | 44 |
