# Supplementary material for: Effectiveness of virtual reality technology in rehabilitation after anterior cruciate ligament reconstruction: A systematic review and meta-analysis
Source: PLoS One. 2025 Mar 3;20(3):e0314766. doi: 10.1371/journal.pone.0314766 (PMC11875343; doi:10.1371/journal.pone.0314766)
Supplement: Attachment — Submitted filename: Response to Reviewers.docx [file pone.0314766.s007.docx]

**Manuscript ID:** PONE-D-24-25139

**Title:** Effectiveness of virtual reality technology in rehabilitation after anterior cruciate ligament reconstruction: A systematic review and meta-analysis

Dear Luciana Labanca,

Thank you very much for your valuable suggestions on our manuscript. These comments will undoubtedly help us further improve our work. We have made the necessary modifications to the manuscript to address your questions and concerns. We resubmitted the revised manuscript to the *PLOS ONE*. Please see the following list of changes and answers to questions.

If there are any questions, please let us know, thanks.

Sincerely yours,

Feng'e Qian, Prof

School of Nursing

Yunnan University of Traditional Chinese Medicine

No. 1076, Yuhua Road, Chenggong District,

Kunming 650500

China

E-mail：FengeQian@outlook.com

**For Reviewer #1**

**Comment 1:** The abstract should succinctly summarize the novel findings and their implications, such as “This study is the first to systematically compare VRT with traditional therapy, providing evidence for the integration of VRT in post-ACLR rehabilitation protocols.”

***Response****:* *Thank you very much for your comment. We have made changes in the Abstract. They are shown in the figure below.*

**Comment 2:** The introduction should clearly state the gap in knowledge that the study addresses. Add a sentence that highlights the lack of consensus on the use of VRT in ACLR rehabilitation, emphasizing the study's contribution to filling this gap.

***Response****:* *Thank you very much for your comment.* *We have made changes in the introduction. They are shown in the figure below.*

**Comment 3:** Clarify the time frames mentioned in this section. It is unclear what is meant by “more than one week” and “one week after surgery.” Specify the exact time points to avoid ambiguity.

***Response****:* *We really appreciate your efforts and comments on our manuscript. Time frames have been specified in the subgroup analysis section. Specifically, “later than 7 days after surgery” and “within 7 days after surgery”.* *As shown in the figure below.*

**Comment 4:** The sensitivity analysis is well-conducted. However, it would be helpful to explain why the study by Gsangaya et al. had such a significant impact on the overall results. Consider adding a sentence or two discussing possible reasons.

***Response****:* *Thank you very much for your comment.* *Possible causes have been added. This is shown in the figure below.*

**Comment 5:** The publication bias section is concise. However, it would be useful to mention the number of studies included in the Egger's test and the effect size.

***Response****:* *Thank you very much for your comment.* *The number of studies included in the Egger's test and the effect size have been mentioned. This is shown in the figure below.*

**Comment 6:** The discussion should provide a more comprehensive synthesis of the findings in relation to existing literature. Discuss the mechanisms behind the observed effects, the clinical implications, and potential future research directions. Also, consider mentioning the limitations of the study and how they might affect the interpretation of the results.

***Response****:* *We really appreciate your efforts and comments on our manuscript.* *A more comprehensive review of the findings has been provided in the context of the existing literature. Mechanisms behind the observed effects, clinical significance and potential future research directions have been discussed. Limitations of the study and how these limitations affect the interpretation of the results have been mentioned. See both the Discussion and Limitations sections of the manuscript for more details.*

**Comment 7:** Some of the statements in the manuscript are not clearly expressed and the quality of the language needs to be improved. Such as: Current: This study aimed to examine the effects of VRT on rehabilitation following ACLR. The findings will help inform decisions regarding the use of VRT in clinical settings.

Current: This study aimed to examine the effects of VRT on rehabilitation following ACLR. The findings will help inform decisions regarding the use of VRT in clinical settings.

Revision Suggestion: This study aimed to evaluate the effects of virtual reality technology on rehabilitation following ACLR, providing insights for its application in clinical settings.

***Response****:* *Thank you very much for your comment.* *The unclear parts of the statement have been improved as shown in the figure below.*

Current: After a selection process, six randomised controlled studies including a total of 387 patients who underwent ACL repair were considered.

Revision Suggestion: Following a rigorous selection process, six randomized controlled trials involving a total of 387 patients who underwent ACL reconstruction were included.

***Response****:* *Thank you very much for your comment.* *This section has been improved. See the figure below.*

Current: The results indicate that VRT had a significant positive effect on various measures.

Revision Suggestion: The findings demonstrate that VRT significantly enhances knee function, walking ability, gait function, and knee muscular strength post-ACLR.

***Response****:* *Thank you very much for your comment.* *This section has been improved. See the figure below.*

**Comment 8:** Ensure consistent use of terms (e.g., always use “virtual reality technology (VRT)” after its first mention).

***Response****:* *Thank you very much for your comment.* *Consistency in the use of terminology has been ensured. Checks have been made to ensure that“acronyms” are always used after the first reference.*

**Comment 9:** Ensure that all references cited in the text are included in the reference list and vice versa. Double-check the formatting of the references to ensure consistency and adherence to the journal's style guide.

***Response****:* *Thank you very much for your comment.* *All cited references have been carefully checked for number, format, and style.*

**For Reviewer #2**

**Comment 1:** Title: really poor and scarce

***Response****:* *Thank you very much for your comment.* *The title has been changed from "Effects of virtual reality technology on anterior cruciate ligament reconstruction: A systematic review and meta-analysis" to "Effectiveness of virtual reality technology in rehabilitation after anterior cruciate ligament reconstruction: A systematic review and meta-analysis".*

**Comment 2:** Methods: report keywords added.

***Response****:* *Thank you very much for your comment.* *We have added the keyword in the Methods.* *As shown in the figure below.*

**Comment 3:** Objectives: any hypothesis

***Response****:* *Thank you very much for your comment.* *We have added the hypothesis in the Objectives section. As shown in the figure below.*

**Comment 4:** Results: too many numbers. hard to read.

***Response****:* *Thank you very much for your comment.* *Appropriate deletions have been made to the figures in the Results section. This is shown in the figure below.*

**Comment 5:** Introduction: little bit too long. Please focus on the topic of your paper.

***Response****:* *We really appreciate your efforts and comments on our manuscript.* *The Introduction has been appropriately restructured by focusing on the theme. For details, see the foreword in the manuscript.*

**Comment 6:** Report current evidences and what are the controversies in literature.

***Response****:* *We really appreciate your efforts and comments on our manuscript.* *Current evidence has been reported as well as controversies in the literature. This is shown in the figure below.*

**Comment 7:** Explain the rationale for your study.

***Response****:* *Thank you very much for your comment.* *The basic principles of virtual reality technology are shown in the figure below.*

**Comment 8:** Finish with aim and hypothesis.

***Response****:* *Thank you very much for your comment.* *We have modified the end of the introduction to include the aims and hypothesis in the content. This is shown in the figure below.*

**Comment 9:** Improve inclusion and exclusion criteria.

***Response****:* *We really appreciate your efforts and comments on our manuscript.* *We have improved the inclusion and exclusion criteria. This is shown in the figure below.*

**Comment 11:** Results: little bit too long.

***Response****:* *Thank you very much for your comment.* *Appropriate adjustments have been made to the Results section to ensure what must be in this study. See the Results section of the manuscript for details.*

**Comment 10:** Discussion: start with main findings of your paper.

***Response****:* *Thank you very much for your comment.* *The Discussion section has been modified to report the main findings of this study from the beginning. This is shown in the figure below.*

**Comment 11:** Report what is new and what this paper add to Current literature.

***Response****:* *We really appreciate your efforts and comments on our manuscript.* *Because this is the first systematic review and meta-analysis of the effectiveness of VRT in post-ACLR rehabilitation. Therefore, this article adds some directions for future research that could be conducted with this patient using VRT. This is shown in the figure below.*

**Comment 12:** Explain how this study can help in clinical daily practice.

***Response****:* *Thank you very much for your comment.* *An explanation of how this research can help clinical daily practice has been included in the article. This is shown in the figure below.*

**Comment 13:** Improve limitations.

***Response****:* *Thank you very much for your comment.* *Improvements have been made to the Limitations section. This is shown in the figure below.*

**Comment 13:** References: add following.

***Response****:* *Thank you very much for your comment.* *We have added these two literatures in the Discussion section, in the text as [30,31]. They are shown in the figure below.*
